# Genomic Insights Into the Lifestyles of Thaumarchaeota Inside Sponges

**DOI:** 10.3389/fmicb.2020.622824

**Published:** 2021-01-11

**Authors:** Markus Haber, Ilia Burgsdorf, Kim M. Handley, Maxim Rubin-Blum, Laura Steindler

**Affiliations:** ^1^Department of Marine Biology, Leon H. Charney School of Marine Sciences, University of Haifa, Haifa, Israel; ^2^Department of Aquatic Microbial Ecology, Institute of Hydrobiology, Biology Centre CAS, České Budějovice, Czechia; ^3^School of Biological Sciences, The University of Auckland, Auckland, New Zealand; ^4^Israel Oceanographic and Limnological Research Institute, Haifa, Israel

**Keywords:** sponge (Porifera), archaea, thaumarchaeota, symbiosis, *Petrosia ficiformis*, *Theonella swinhoei*, *Hymedesmia (Stylopus) methanophila*

## Abstract

Sponges are among the oldest metazoans and their success is partly due to their abundant and diverse microbial symbionts. They are one of the few animals that have Thaumarchaeota symbionts. Here we compare genomes of 11 Thaumarchaeota sponge symbionts, including three new genomes, to free-living ones. Like their free-living counterparts, sponge-associated Thaumarchaeota can oxidize ammonia, fix carbon, and produce several vitamins. Adaptions to life inside the sponge host include enrichment in transposases, toxin-antitoxin systems and restriction modifications systems, enrichments previously reported also from bacterial sponge symbionts. Most thaumarchaeal sponge symbionts lost the ability to synthesize rhamnose, which likely alters their cell surface and allows them to evade digestion by the host. All but one archaeal sponge symbiont encoded a high-affinity, branched-chain amino acid transporter system that was absent from the analyzed free-living thaumarchaeota suggesting a mixotrophic lifestyle for the sponge symbionts. Most of the other unique features found in sponge-associated Thaumarchaeota, were limited to only a few specific symbionts. These features included the presence of exopolyphosphatases and a glycine cleavage system found in the novel genomes. Thaumarchaeota have thus likely highly specific interactions with their sponge host, which is supported by the limited number of host sponge species to which each of these symbionts is restricted.

## Introduction

Sponges (phylum Porifera) are sessile, soft-bodied invertebrates that inhabit marine and freshwater environments around the globe (Van Soest et al., 2012). As highly efficient filter feeders, they are a crucial link between the pelagic and benthic environments (Yahel et al., 2003; de Goeij et al., 2013). Their success has been attributed to their microbial symbionts (Taylor et al., 2007) that can make up to 38% of their tissue volume (Vacelet and Donadey, 1977). Sponge symbionts, including bacteria and archaea, can be transmitted both horizontally and vertically (Steger et al., 2008; Björk et al., 2019). The symbionts support their host in various ways. They produce secondary metabolites that are likely involved in the chemical defense of their host against predators, fouling, and pathogens (Waters et al., 2014; Mori et al., 2018); remove waste products produced by their eukaryotic host, such as ammonia (Moeller et al., 2019); contribute nutrients (Erwin and Thacker, 2008; Mohamed et al., 2008; Rubin-Blum et al., 2019); and might even be involved in skeleton formation (Uriz et al., 2012). Given that sponges are one of the earliest-branching multi-cellular animals (Metazoa) (Feuda et al., 2017), these symbiotic interactions are considered ancient.

The microbial communities in sponges are often complex (Thomas et al., 2016) and comprise more than 60 different bacterial and archaeal phyla (Moitinho-Silva et al., 2017b). In many cases, these symbionts fall into phylogenetic clades made up entirely of sequences derived from sponges (and sometimes corals) suggesting highly specific relationships (Simister et al., 2012; Taylor et al., 2013). Comparative analyses of metagenomes from sponge-associated and seawater communities found symbiont consortia to be enriched in features such as transposable elements, defense mechanisms (e.g., CRISPR-Cas systems) and eukaryote-like proteins, potentially involved in symbiont recognition and interaction (Thomas et al., 2010; Fan et al., 2012; Horn et al., 2016; Slaby et al., 2017). Studies on individual bacterial clades revealed several additional features that differ between sponge symbionts and their free-living counterparts. The widespread sponge-associated cyanobacteria *Candidatus* Synechococcus spongiarum have more streamlined genomes than their close free-living relatives, lost genes encoding low molecular weight peptides involved in stabilizing and protecting the photosynthesis apparatus, and have fewer genes related to oxidative stress protection and signal transduction (Gao et al., 2014; Burgsdorf et al., 2015). Sponge symbionts of the Alphaproteobacteria family Rhodospirillaceae lack genes related to chemotaxis and motility compared to free-living bacteria from the same family, but have more genes related to the biosynthesis of secondary metabolites and cell detoxification (Karimi et al., 2018).

Bacteria usually dominate sponge microbial communities, but archaea are regularly found in sponge hosts and can make up substantial parts of the community (e.g., 24% of the microbial community in the Great Barrier reef sponge *Ianthella basta*) (Moeller et al., 2019), and in some cases even dominate the communities (e.g., up to 63% in the microbial community of the sponge *Axinella mexicana*) (Preston et al., 1996). A comparison of the archaeal sponge community from hosts living in shallow waters and the deep-sea indicated that sponge-associated archaea appear to be much more abundant (up to three orders of magnitude greater) in the deep-sea (Steinert et al., 2020). Most archaeal sponge symbionts are Thaumarchaeota, with some belonging to sponge-specific clades (Simister et al., 2012). Thaumarchaeota are found in sponge larvae (Steger et al., 2008) and also in adult sponges, that acquire their symbionts horizontally (Burgsdorf et al., 2014), indicating the same variability in transmission routes as found in bacterial sponge symbionts (Sipkema et al., 2015). Thaumarchaeota are well-known ammonia-oxidizers (Pester et al., 2011; Baker et al., 2020) making them a key player in sponge nitrogen metabolism and nitrogenous waste removal. While their role in the sponge nitrogen cycle is well established based on *in situ* physiology measurements (Bayer et al., 2008; Radax et al., 2012a; Moeller et al., 2019), *amoA* gene expression studies (López-Legentil et al., 2010; Zhang et al., 2014) and more recently metagenome (Moitinho-Silva et al., 2017a; Moeller et al., 2019; Zhang et al., 2019), metatranscriptome (Radax et al., 2012b; Moitinho-Silva et al., 2017a) and metaproteome analyses (Moeller et al., 2019), less is known about the genomic adaptations of Thaumarchaeota to life inside sponges.

The first published genome of a thaumarchaeal sponge symbiont is that of *Cenarchaeum symbiosum*, hosted by the demosponge *Axinella mexicana* (Hallam et al., 2006). Recently, additional genomes of archaeal symbionts have become available, including those hosted by deep-sea and glass sponges (Tian et al., 2016, 2017; Moitinho-Silva et al., 2017a; Moeller et al., 2019; Zhang et al., 2019). Only a limited number of studies compared the genomes of symbiotic and free-living Thaumarchaeota (Moeller et al., 2019; Zhang et al., 2019). Moeller et al. (2019) compared a metagenome-assembled thaumarchaeal genome (MAG) from the sponge *Ianthella basta* to those of three other sponge-associated and several free-living Thaumarchaeota. Zhang et al. (2019) compared the MAGs of three sponge-associated *Nitrosopumilus* species with those of their free-living relatives. Both studies found that the sponge symbiont genomes had elevated GC content. These symbiotic Thaumarchaeota are likely mixotrophs, due to the presence of a branched-chain amino acid transporter and are enriched in mechanisms for defense against phages, such as restriction-modification systems. Sponge-associated Thaumarchaeaota also encode eukaryotic-like proteins, which are absent from free-living Thaumarchaeota, that are assumed to be involved in interactions with the sponge hosts (Moeller et al., 2019; Zhang et al., 2019). Here, we aim to expand our knowledge of the genomic repertoire in sponge-associated Thaumarchaeota. We analyzed three new MAGs of sponge-associated Thaumarchaeota, together with the nine previously reported sponge-associated archaea genomes, and the high-quality (>95% completeness) genomes of twelve free-living Thaumarchaeota.

## Materials and Methods

### Sponge Sampling and DNA Isolation

Samples of the sponges *Theonella swinhoei* and *Petrosia ficiformis* were collected by SCUBA from the Gulf of Aqaba, Red Sea (29°29′N 34°54′E) at 25 m, Israel on July 31st, 2012 and the Achziv nature marine reserve (33°00′N 35°02′E) at 20 m, Israel on May 5th, 2013, respectively, and transported on ice to the laboratory for further processing (*T. swinhoei* within 20 min to IUI-Eilat and *P. ficiformis* within 2 h to University of Haifa, respectively). Cortex tissue of *P. ficiformis* and both cortex and endosome of *T. swinhoei* were used for microbial cell enrichment, followed by DNA extraction. Microbial cell enrichment by a series of filtration and centrifugation steps and DNA extraction followed previously described methods (Thomas et al., 2010).

The asphalt-encrusting deep-sea demosponge *Hymedesmia* (*Stylopus*) *methanophila* was collected using the ROV QUEST 4,000 m (Marum, Bremen) operated from the research vessel Meteor during the M114-2 cruise to Campeche Knolls, Southern Gulf of Mexico, in March 2015 (Rubin-Blum et al., 2019). The specimen was collected from the Chapopote knoll (21°54′ N; 93°26′ W at 2,925 m. The collection site is described in detail elsewhere (Sahling et al., 2016). Upon retrieval, the sponge tissue was removed from the underlying asphalt with a scalpel, fixed in RNAlater (Sigma^®^, Steiheim, Germany) according to the manufacturer’s instructions and stored at −80°C. DNA was extracted with the AllPrep DNA/RNA Mini Kit (Qiagen, Hilden, Germany) following the manufacturer’s instructions.

### Shotgun Sequencing, Assembly, and Taxonomic Binning

*T. swinhoei* and *P. ficiformis* microbiomes were sequenced using an Illumina HiSeq2000 platform (2 × 100 bp, paired-end) at the Institute for Genomics and Systems Biology’s Next Generation Sequencing Core (IGSB-NGS, ANL) at the University of Chicago. Libraries were generated using the TruSeq DNA standard protocol and pooled for sequencing using the Illumina HiSeq2000 platform. 69 and 129 million 100 bp sequences were generated from *T. swinhoei* and *P. ficiformis*, respectively. Sequence quality was assessed and low-quality reads (q = 3) were trimmed using the FASTX-Toolkit 0.0.13.2^1^. Sequence data sets were assembled *de novo* using IDBA-UD version 1.1.0 (Peng et al., 2012) with a kmer range of 50–70, and a step size of 5, following empirical tests. To assign contigs into genome bins, genes on contigs ≥2 kb long were predicted using Prodigal with the metagenome option (Hyatt et al., 2010, 2012). For each contig, we determined the GC content, coverage, and the taxonomic affiliation based on the best hit for each predicted protein in the Uniref90 database (accessed September 2013; Suzek et al., 2007) following UBLAST searches (usearch64; Edgar, 2010). Contigs were assigned to MAGs using these data, as well as emergent self-organizing maps (ESOM) based analysis of fragment tetranucleotide frequencies (Dick et al., 2009), as detailed by Handley et al. (2013). Among the metagenome-assembled genomes (MAGs) obtained from *T. swinhoei* was one archaea genome containing 158 contigs and 1,859,711 bp (coverage 65×). *P. ficiformis* contained two archaea genomes: one with 108 contigs and 1,791,570 bp and a second one with 161 contigs and only 521,474 bp. These two genomes (labeled bin A and B) were easily distinguishable based on their coverage (493× vs. 38×).

Genomic DNA libraries for *Hymedesmia* (*Stylopus*) *methanophila* were generated with the DNA library prep kit for Illumina (BioLABS, Frankfurt am Main, Germany) and sequenced on the Illumina HiSeq 2500 platform at the Max Planck Genome Centre (Cologne, Germany). 12.5 million 250 bp paired-end metagenomic reads were generated, while the remaining 15.5 million were generated as 150 bp paired-end reads. The metagenome was assembled with IDBA-UD (Peng et al., 2012) following decontamination, quality filtering (Q = 2) and adapter-trimming of the reads with BBDuk tool from the BBMap suite (Bushnell B)^2^. The archaeal MAG was binned based on genome coverage, GC content and taxonomic affiliation with genome-bin-tools (Seah and Gruber-Vodicka, 2015). The MAG was reassembled with Spades V3.10 (Bankevich et al., 2012) using a maximum k-mer length of 127, following re-mapping of Illumina reads to the bins using BBMap with 0.98 minimum identity. Contigs of <2 kbp were removed and the resulting bin was used in the analysis. Hereafter, we refer to this MAG as *Ca*. Nitrosopumilus sp. ESC.

### Genome Dataset

In addition to the three sponge-associated archaeal genomes generated in this study, 20 previously published genomes were downloaded from NCBI and IMG for comparisons. These included 8 sponge symbiont genomes: *Crenarchaeum symbiosum* A (Hallam et al., 2006), *Ca.* Nitrosopumilus sp. LS AOA (Tian et al., 2016), *Ca.* Nitrosopumilus sp. Nsub (Tian et al., 2017) the most complete genome of each of *Ca.* Nitrosopumilus hexadellus, *Ca*. Nitrosopumilus detritiferus, and *Ca*. Cenporiarchaeum stylissum (Zhang et al., 2019), *Ca*. Nitrosopumilus cymbastelus (Moitinho-Silva et al., 2017a), *Ca*. Nitrosospongia ianthallae (Moeller et al., 2019). In addition, 12 free-living Thaumarchaeota genomes were retrieved that belong to the Nitrosopumilaceae family (5 from marine sediment, 5 from marine pelagic, 2 from soil) (see Supplementary Table S1 for details). The selection of free-living genomes was based on passing an estimated genome completeness threshold of >95% based on 46 single copy COGs (see below for details of assessment).

### Completeness Estimation, and Functional Annotation

All 23 genomes were uploaded to RAST (Aziz et al., 2008; Overbeek et al., 2014). Open reading frames (ORFs) were identified with the classic RAST algorithm and SEED annotations were obtained. Protein fasta files of predicted ORFs were downloaded and protein domains (PFAM) (Finn et al., 2010) and clusters of orthologous groups (COGs) (Tatusov et al., 2003) were annotated through the WebMGA annotation tool (Wu et al., 2011) with an e-value cutoff of 0.001. COGs were identified by rpsblast 2.2.15 searches against the NCBI COG database 2/2/2011. In the case of multiple COGs, only the best hit was retained. COGs were assigned to COG classes according to Galperin et al. (2015). PFAMs were assigned with hmmscan 3.0 and the Pfam database 24.0.

To select genomes for analysis, genome completeness was first determined by comparing the annotated COGs to a list of 53 essential single-copy COGs previously described to be present in all archaea (Puigbò et al., 2009). After the exclusion of COG0455 that was absent in all analyzed Thaumarchaeota genomes, and six COGs that were present in multiple copies in more than half of the analyzed genomes (present in 17–28 genomes in multiple copies), 46 COGs remained for completeness estimation. Only one of these COGs was present more than once in two or more genomes (5 times). Only sponge-associated Thaumarchaeota genomes with >90% completeness (>41 of the 46 COGs present) and free-living ones with >95% completeness (>43 COGs) were used in the analyses. Of the four newly generated sponge symbiont MAGs, three were retained. The rarer archaeal MAG (bin B) from *P. ficiformis* contained only 19 of the 46 single-copy COGs (41.3% completeness) and thus was excluded from further analysis. For the selected genomes, we then assessed genome completeness and contamination using CheckM (Parks et al., 2015). Completeness results of both methods are reported in the Supplementary Table S1 and details for the 46 COGs in Supplementary Table S2.

### Comparative Genomic Analyses

COG annotations of MAGs were compared to identify functions specific to symbiotic and free-living Thaumarchaeota. To test for significant differences in COG and COG class relative abundances between free-living and sponge-associated Thaumarchaeota we used a two-tailed t-test implemented in STAMP v.2.1.3 (Parks et al., 2014). *P*-values were corrected using false discovery rate corrections according to Benjamin-Hochberg. COGs associated with multiple classes were added to each of the classes. COGs and COG classes with corrected *p*-values <0.05 were considered enriched. We determined enrichment in specific groups of COGs and Pfams with similar functions were tested in the same way using a two-tailed *t*-test and FDR correction.

### Taxonomic Classification and Phylogenetic Analyses

A phylogenomic tree for the dataset of 11 symbiotic archaeal, 12 free-living archaeal and 4 outgroup genomes was based 182 single copy marker genes and constructed as follows: A concatenated alignment of protein sequences was generated using PhyloPhlAn2 (Segata et al., 2013). The alignment was then used to infer maximum-likelihood trees with RAxML (Stamatakis, 2014) with the PROTCATLG model of evolution and 1000 bootstrap replications. The best tree was visualized with iTOL (Letunic and Bork, 2019).

Near full-length 16S rRNA gene sequences were obtained from the RAST annotated genomes of the free-living and sponge-associated archaea, except bin A from *P. ficiformis*, which contained a 64 bp fragment at the end of a contig. We generated a targeted small subunit rRNA gene assembly from the *P. ficiformis* metagenome using EMIRGE (Miller et al., 2011) and a 16S rRNA gene clone library from the same DNA as used for the metagenome (see Supplementary Material for details). The resulting 11 sequences were used in the phylogenetic analyses.

These sequences were analyzed together with representative sequences of five previously established sponge-specific Thaumarchaeota clusters and the closest related non-host associated sequences [SC174-178 from Simister et al. (2012) as well as the closest environmental sequences for the sponge symbionts as determined by BLAST search (Altschul et al., 1990)]. The 16S rRNA gene sequences of three group I.1b thaumarchaota (*Ca.* Nitrosphaera gargensis Ga92, *Ca*. N. evergladensis SR1, and *Ca*. N. viennensis EN76) were used as outgroup. Sequences were aligned with the SINA aligner version 1.2.11 (Pruesse et al., 2012) and the resulting alignment was manually improved. A maximum likelihood tree was calculated in MEGA X (Kumar et al., 2018) using the Kimura-2-parameter substitution model with a portion of invariant sites and a gamma-shaped distribution of mutation rates, which was the best model for the data according to model test implemented in MEGA X. Sites with >5% ambiguous or missing data were omitted. The best tree was found using a heuristic search with the NNI algorithm starting from an initial Neighbor-Joining tree. The robustness of the tree was tested using 500 bootstraps replicates.

All genomes were also classified with GTDB-Tk v0.3.2 (Parks et al., 2018) with the default parameter classify_wf to determine the taxonomy of the genomes.

### The Relative Abundance of Thaumarchaeal Symbionts in Different Sponge Species

To analyze the abundance of the symbiotic Thaumarchaeota in diverse sponge species from around the world, we searched the Sponge Microbiome Project (SMP) (Thomas et al., 2016; Moitinho-Silva et al., 2017b) part of the Earth Microbiome Project (EMP^3^). The project contains 16S rRNA gene amplicon data from the hypervariable V4 region. We used the 16S rRNA gene sequences from the MAGs as a query for BLASTn 2.2.30 + (Altschul et al., 1990). Sequences matching our genomes with alignment length ≥95 bp and a maximum of 1 mismatch (99% similarity) were used to determine the relative abundance of the symbiotic Thaumarchaeota among the SMP samples. Sequences with relative abundances below 0.1% in the samples were not used in the analysis. The binomial (presence/absence) *p*-values enrichment (Moitinho-Silva et al., 2017b) among various sponge species and environmental samples were calculated and corrected as described previously (Burgsdorf et al., 2019). *p*-values were corrected for multiple testing using the p.adjust() R function and the false-discovery (FDR) correction. Samples with corrected *p*-values of ≤0.05 were considered enriched. Only environmental samples and sponge species with at least three replicates were included in the analysis. Only samples from sponges collected directly from their habitat were included. Samples of unhealthy or manipulated sponges and samples with unclear taxonomy (e.g., “Porifera”) were excluded. The final dataset included 2,131 tissue samples from of 180 sponge species, collected in 33 countries, as well as 305 seawater and 54 marine sediment samples. Boxplots were created using the ggplot2 package in Rstudio.

In addition, we mapped reads from 55 sponge metagenomes obtained 13 sponge species against the genomes of the sponge-associated Thaumarchaeota. All but six metagenomes were quality trimmed (quality threshold 20) with the reformat tool from the BBtools package^4^. Of the remaining six, three metagenomes (IMG accession numbers 3300003175, 3300003251, 3300003254) were quality trimmed as described in Burgsdorf et al. (2015) and the other three (SAMN11333419, SAMN11333441, SAMN12828169) according to Burgsdorf et al. (2019). The quality-filtered and -trimmed reads were mapped against the genome sequences of the sponge-associated Thaumarchaeota using bbmap tool v 37.62 from the BBtools package with default kmer length of 13 and minimum percentage identity cutoff of 95%.

### Data Deposition

Metagenomes of *T. swinhoei* and *P. ficiformis* were submitted to the Integrated Microbial Genomes (IMG) database (Markowitz et al., 2012)^5^ and are accessible via the accession numbers 3300003175 and 3300003254, respectively. The draft genomes of Thaumarchaeon TS and *Ca*. Nitrosopumilus sp. Pfa have been deposited in GenBank under JAEFDA000000000 and JAEFCZ000000000 accession numbers, respectively. The metagenome of *Hymedesmia* (*Stylopus*) *methanophila* and the draft genome of its thaumarchaeal symbiont were deposited in GenBank under the project number PRJNA475438. 16S rRNA sequences from the clone library of *P. ficiformis* were deposited under accession numbers MT876199-MT876208.

## Results and Discussion

### General Genomic Information

The estimated genome completeness based on CheckM ranged from 75.6 to 87.5% with 1.2–8.9% potential contamination for the sponge-associated and 84.6–88.9% with 3.0–9.5% potential contamination for the analyzed free-living Thaumarchaeota genomes (Supplementary Table S1). Estimated genome sizes and the number of predicted genes did not differ significantly between genomes from the sponge-associated and free-living archaea (two-tailed *t*-tests, *p* > 0.05). This is in-line with the general observation that genome reduction in symbiotic archaea is not seen or at least not to the same extent as in symbiotic bacteria (Kellner et al., 2018).

One of the few previously identified general patterns for sponge-associated archaeal genomes is a higher GC content compared to free-living Thaumarchaeota (Moeller et al., 2019; Zhang et al., 2019). Our analysis confirmed the overall higher %GC of the symbiotic Thaumarchaeota but also indicated that this pattern is not universal. Apart from the soil-associated *Nitrosotenius chungbukensis* MY2, which had a relatively high GC content (41.8%), the GC content of the analyzed free-living Thaumarchaeota ranged from 32.5 to 34.2%. Seven of the eleven sponge-associated had GC content between 47 and 67%, the exception being *Ca*. Nitrosopumilus cymbastelus (38.4%) and all three deep-sea sponge symbionts (31.4–33.3%). As the GC content is shaped both by phylogeny and the environment (Reichenberger et al., 2015), the Thaumarchaeota with elevated GC content are likely true symbionts and were not transient as a result of the filter feeding of the sponge host. The inclusion of the here generated genome of the thaumarchaeal symbiont from the deep-sea sponge *Hymedesmia* (*Stylopus*) *methanophila* suggests that low GC content is a general feature among deep-sea sponge-associated Thaumarchaeota. This is further supported by the recently published thaumarchaeal genomes from the deep-sea glass sponge *Vazella pourtalesii*, which had GC content between 31.8 and 40.4% (Bayer et al., 2020). The general features of the analyzed genomes are summarized in Supplementary Table S1.

### Taxonomic Identity and Phylogeny

**FIGURE 1:**
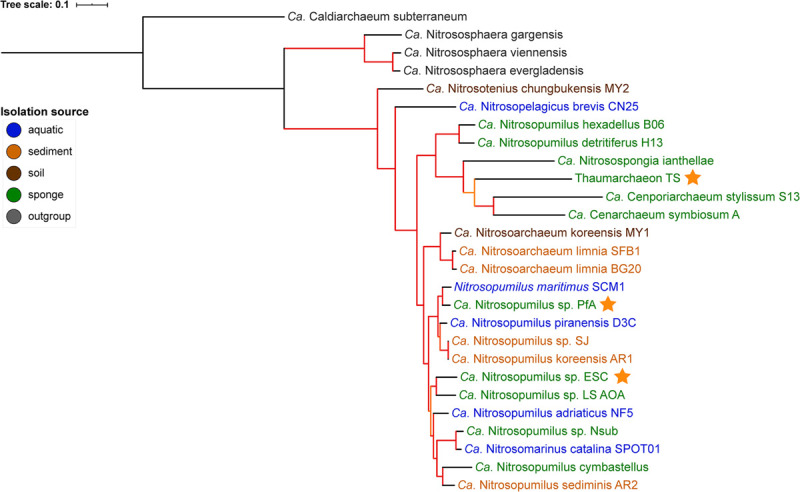
Phylogenomic tree of free-living and sponge-associated Thaumarchaeota. Origin is indicated by font color. Genomes obtained in this study are indicated by an orange star. The tree was calculated using maximum likelihood with the PROTCATLG model of evolution and is based on 182 single copy marker genes (see “Materials and Methods” section for details). Branch colors indicate bootstrap support based on 1,000 repetitions: orange 87–89%, red > 97%. The tree was rooted with *Ca*. Caldiarchaeum subterraneaum (black branch).

GTDB-tk placed the genomes generated from *P. ficiformis* and *H.* (*S.*) *methanophila* as new species within the genus *Nitrosopumilus* with an average nucleotide identity (ANI) of <88% to the closest species in GTDB (Supplementary Table S3). Their placement within the *Nitrosopumilus* genus was supported by the 16S rRNA gene tree (Supplementary Figure S1) and the phylogenomic tree (Figure 1). In the 16S rRNA gene tree, all *P. ficiformis* derived sequences grouped together. Hence, we refer to the two novel genomes as *Ca*. Nitrosopumilus sp. PfA and ESC, respectively. They join five previously available sponge-associated genomes in the *Nitrosopumilus* genus. The third new symbiont genome, obtained from *T. swinhoei*, had an ANI of 76.6% to the closest match in GTDB, the sponge symbiont *Cenarchaeum symbiosum*. It was assigned as new species within the *Cenarchaeum* genus by the GTDB-tk classification algorithm but a new genus by pplacer taxonomy (Supplementary Table S3). Given this inconsistency, the fact that the genus *Cenarchaeum* is represented by just one species, and that our MAG from *T. swinhoei* did not group in a well-supported clade with *C. symbiosum* neither in the phylogenomic nor the 16S rRNA tree, we refrained from assigning taxonomy to our MAG and refer to it as Thaumarchaeon TS.

None of the three novel genomes were part of previously identified sponge-specific clusters (Simister et al., 2012) in the 16S rRNA gene tree. Of the previously available genomes, *Ca*. Nitrosopongia ianthellae, *Ca.* Nitrosopumilus hexadellus B06 and *Ca*. Nitrosopumilus detritiferus H13 might be part of the sponge-specific cluster (SC) 174 and *Ca*. Cenporiarchaeum stylissum S13 of SC176. These results are in line with previous reports (Moeller et al., 2019; Zhang et al., 2019) and indicate a high degree of specificity of their association with sponges. A major difference between the 16S rRNA tree and the phylogenomic tree was the placement of the clade containing *Ca.* Nitrosopumilus hexadellus B06, *Ca*. Nitrosopumilus detritiferus H13, and *Ca*. Nitrosospongia ianthellae. The taxonomy algorithm of GTDB-tk placed the former two within *Nitrosopumilus* and the latter outside of it (see Supplementary Table S3). In the 16S rRNA tree they all grouped within the *Nitrosopumilus* genus, while in the phylogenomic tree they grouped outside of the clade containing the *Nitrosopumilus* and *Nitrosoarchaeum* genera, a placement consistent with previous analysis (see Supplementary Figure S1B in Zhang et al., 2019).

### Distribution of Thaumarchaeal Symbionts in Sponge and Environmental Samples

To investigate how widespread the analyzed thaumarchaeal symbionts are in sponges, we used their 16S rRNA sequences as queries for BLAST searches against the Sponge Microbiome Project (SMP), an amplicon database of the V4 region of the 16S rRNA gene from >3,500 sponge specimen collected worldwide as well as seawater and sediment samples collected next to the sponges (Moitinho-Silva et al., 2017b). The symbionts displayed different specificity ranging from generalists (found in a wide range of sponge species and environmental samples) to specialists (which live in obligate association with a small number of sponge species).

**FIGURE 2:**
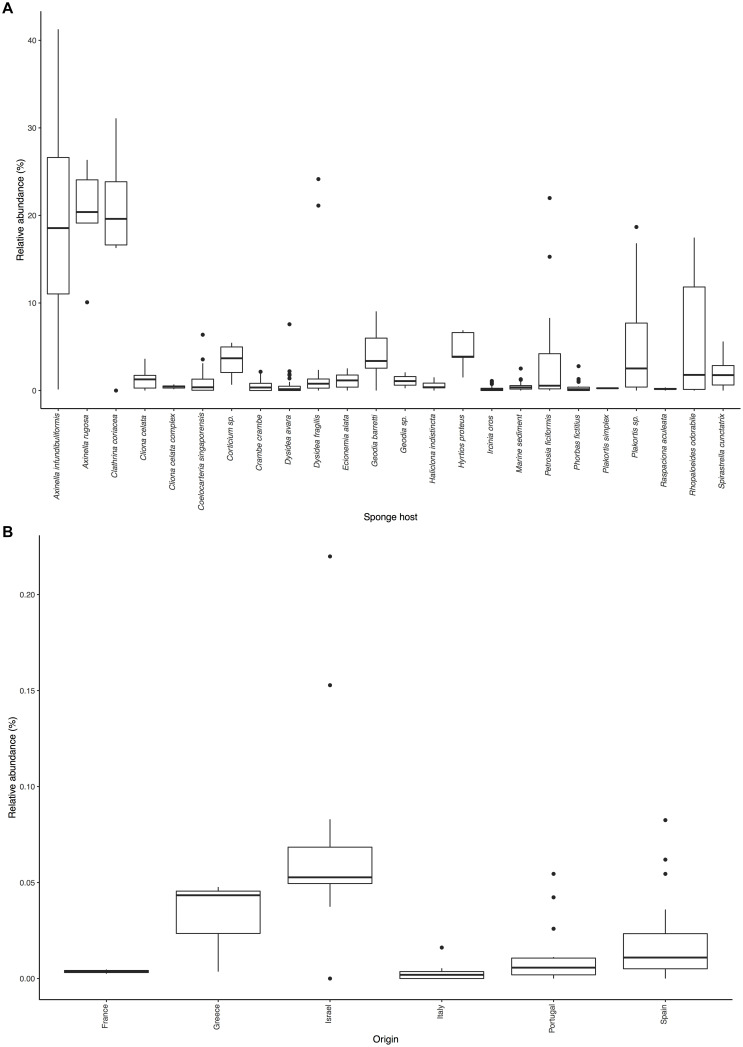
Distribution of *Ca*. Nitrosopumilus sp. PfA.: **(A)** Sponge species and environmental samples with significant association. **(B)** Presence in samples of host sponge *Petrosia ficiformis* from different locations.

*Ca*. Nitrosopumilus sp. PfA, Nsub and LS AOA (which have an identical 16S rRNA gene sequence in the V4 region used in the SMP) as well as *Ca*. Nitrosopumilus sp. ESC and *Ca*. N. hexadellus B06 are considered by us to be generalists. They were significantly associated with 23 sponge species (average relative abundance ranging from 0.2 to 20.7%) and were also found in marine sediment samples (0.5%) (Binominal test, FDR-corrected *p*-value <0.1) (Figure 2A and Supplementary Table S4). We likely underestimated their distribution due to the limited power of the statistical test (e.g., presence in 3 of 3 sponge samples was not significant). Given that the *Nitrosopumilus* species are highly related to each other, this wide-spread distribution is not surprising. To further differentiate them from one another, we performed a read recruitment analyses using 55 sponge metagenomes from 13 sponge species. *Ca*. Nitrosopumilus sp. PfA, Nsub and LS AOA clearly differed in this analysis, as most reads were recruited from sponge metagenomes of the host species (Supplementary Table S5). However, they were also detected in other sponge species albeit at a lower level (e.g., one to two orders of magnitude less than in their host sponge) confirming a broad distribution.

Even with the low resolution of the short sequences present in the SMP, we were able to detect an effect of biogeography on the abundance of *Ca*. Nitrosopumilus sp. PfA (Figure 2B). It was most abundant in samples from Israel (average relative abundance 7.1%), from where its genome was recovered (Supplementary Table S6). Its abundance was lower in samples from Portugal (average relative abundance 1.2%), Spain (2.1%), France (0.4%), Italy (0.3%) and Greece (3.2%). The recruitment analysis of the whole genome supports this trend as *Ca*. Nitrosopumilus sp. PfA recruited a higher proportion of reads from metagenomes of sponge specimens collected from Israel than from a Greek specimen (Supplementary Table S6). This is in line with the previous observation that the whole microbial community associated with its host sponge *P. ficiformis* is heavily influenced by biogeography (Burgsdorf et al., 2014).

The other six symbionts (*Ca*. Cenporiarchaeum styllisum S13, *Ca*. Nitrosospongia ianthaella, *Cenarchaeum symbiosum* A, Thaumarchaeon TS, *Ca*. Nitrosopumilus detritiferus H13, *Ca*. Nitrosopumilus cymbastellus) were significantly associated with up to 13 sponge species and not with environmental samples (Supplementary Table S4). They also recruited only a small proportion of reads from most sponge metagenomes apart from their host sponge (Supplementary Table S5). Hence, we regard them as specialists. The degree of specificity varied: *Ca*. Nitrosopumilus detritiferus H13 and *Cenarchaeum symbiosum* A were not significantly associated with any sponge species indicating that they are highly specific symbionts of their host sponges, *Hexadella* cf. *dedritifera* and *Axinella mexicana*, respectively. Both sponge species were not present in the SMP dataset. Host phylogeny had a clear effect on the distribution of *Ca*. Cenporiarchaeum styllisum S13 and *Ca*. Nitrosospongia ianthaella. The former was only significantly associated with sponges of the Agelasida C6 clade [Figure 2A in Morrow et al. (2012) shows the phylogeny of some of these host sponge species], while the latter was significantly associated only with *Ianthella* basta (22.3%), from which it was recovered, and another Verongiida sponge (16.3%) (Supplementary Table S4).

The differentiation between specialist and generalist did not correlate with the symbiont taxonomy as *Ca*. N. detritiferus H13 is highly specific to its host, while the closely related *Ca*. N. sp. hexadellus B06 is a generalist. However, it is possible that *Ca*. N. detritiferus H13 was not found in any sample due to the strict thresholds in our analysis and may be present in very low abundance in other sponges.

### Carbon and Nitrogen Metabolism

Except for a recently discovered basal lineage (Aylward and Santoro, 2020), Thaumarchaeota can fix carbon via the highly energy-efficient 3-hydroxypropionate/4-hydroxybutyrate pathway (Könneke et al., 2014; Baker et al., 2020). The pathway was present in all Thaumarchaeota genomes analyzed here (Supplementary Table S8). Therefore, symbiotic Thaumarchaeota could potentially contribute to the sponge’s carbon demands by transferring fixed carbon to the host.

Thaumarchaeota are also well known for their ability to perform ammonia oxidation (Baker et al., 2020). All genomes contained an ammonia transporter and all, but one had *amoA*, *amoB* and *amoC* genes (Supplementary Table S11). Only the newly obtained genome of Thaumarchaeon TS lacked *amoC*. The *amoA* gene and a hypothetical gene (*amoX*) of the ammonia monooxidase gene cluster were found at the end of one contig and a potential *amoB* at the end of another contig. Assuming the same gene synteny (*amoA*-*amoX*-*amoC*-*amoB*) as in most Nitrosopumilaceae (Park et al., 2014), this suggests that *amoC* was not assembled, and Thaumarchaeon TS is likely able to oxidize ammonia. Previous metatranscriptomic and -proteomic studies reported that ammonia oxidation and ammonia transporter genes are expressed in sponges (Liu et al., 2012; Radax et al., 2012b; Moitinho-Silva et al., 2017a; Moeller et al., 2019). Hence, Thaumarchaeota symbionts are likely involved in the removal of ammonia, a waste product of their sponge hosts (Jiménez and Ribes, 2007; Bayer et al., 2008; Hoffmann et al., 2009; Morley et al., 2016).

Some sponges excrete urea as a nitrogenous waste product (Morley et al., 2016). Eight sponge symbionts (and 3 free-living Thaumarchaeota) had a complete set of urease genes including a potential regulator and thus have the potential to produce ammonia from urea. Most of them encoded a transporter of the sodium:solute symporter family, which likely works as urea transporter in Thaumarchaeota (Offre et al., 2014; Carini et al., 2018). A cultured free-living Thaumarchaeota encoding the urease gene cluster was previously shown to grow on urea as a sole form of energy and nitrogen (Carini et al., 2018). Hence the eight sponge symbionts studied here might be able to live off sponge-derived urea as sole energy and nitrogen source.

**FIGURE 3:**
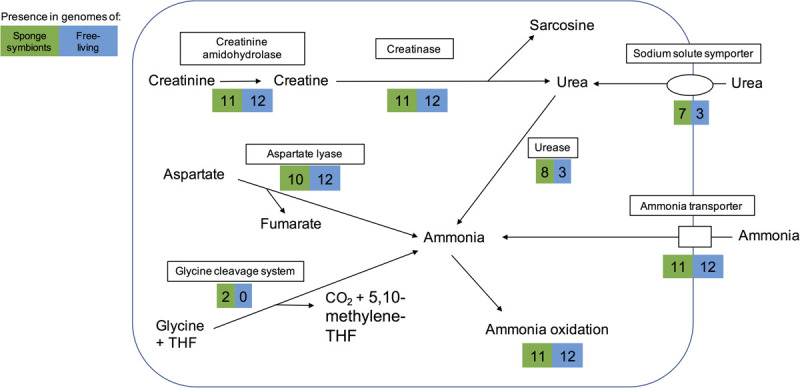
Pathways for generating ammonia in the sponge-associated and free-living Thaumarchaeota. Boxes indicate proteins or pathways. Numbers inside colored boxes represent number of genomes found with the pathway or protein. The maximum number of sponge-associated and free-living MAGs is 11 and 12, respectively. THF, tetrahydrofolate.

Ammonia can also be obtained from other sources such as cyanate, creatinine, and aspartate. None of the genomes analyzed here encoded a cyanase, which catalyzes the reaction from cyanate to ammonia. However, as previously reported for some sponge-associated Thaumarchaeota (Moitinho-Silva et al., 2017a; Moeller et al., 2019), we found that all encoded a creatinine amidohydrolase (annotated either by COG1402 or PF02633). This likely enables the sponge symbionts to produce creatine from sponge-derived creatinine. In contrast to previous reports (Moitinho-Silva et al., 2017a; Moeller et al., 2019), we found a potential creatinase domain in all genomes, based on PFAM annotations (PF01321) and BLAST searches. Creatinase converts creatine into urea and sarcosine. In addition, most genomes also had a Xaa-Pro aminopeptidase (COG0006), which has been hypothesized to work as a functional analog to creatinase (Moitinho-Silva et al., 2017a). Eight sponge symbionts and three free-living Thaumarchaeota could potentially use the resulting urea to produce ammonia (Figure 3). The alternative pathway of creatinine degradation to sarcosine and ammonia via cytosine deaminase (COG0402), *N*-methylhydantoinase (COG0145), and *N*-carbamoylsarcosine amidase (PF00857) appeared to be incomplete in all but one genome. Cytosine deaminase (COG0402) was only missing from the sponge symbiont Thaumarchaeon TS. *N*-methylhydantoinase (COG0145) was present in three sponge-associated genomes and eight free-living ones. Two genomes (Thaumarchaeon TS, *Ca*. Nitrosopumilus sediminis AR2) had urease genes as well, giving them potentially two different routes from creatinine to ammonia. All other genomes with COG0145 lacked urease genes, while the deep-sea sponge symbiont *Ca*. Nitrosopumilus sp. LS AOA lacked both COG0145 and urease genes. *N*-carbamoylsarcosine amidase (PF00857 part of the isochorismatase family), which catalyzes the conversion to sarcosine, was only present in the sediment-dwelling *Ca.* Nitrosopumilus sediminis AR2. It is possible that other Thaumarchaeota use an alternative enzyme that has not been annotated. All but one genome encoded aspartate-ammonia lyase, which lyses L-aspartate to fumarate and ammonium (COG1027), suggesting the ability to produce ammonium from aspartate. Hence, we conclude that sponge symbionts have several ways to obtain ammonia for oxidation and energy production (Figure 3).

For the sponge-associated Thaumarchaeota, nitrogen availability is likely stable, based on the low copy numbers of nitrogen regulatory protein P-II-encoding genes (PF00543) in *Cenarchaeum symbiosum* and *Ca*. Nitrosospongia ianthallae (Moeller et al., 2019). Our analysis indicated that this seems to be true for most sponges and their symbionts. The nitrogen regulatory protein P-II and a negative transcriptional regulator of the NmrA-like family (PF05368) were both more common in the free-living Thaumarchaeota than the sponge-associated ones (Supplementary Table S11). An exception was the newly obtained genome of *Ca*. Nitrosopumilus sp. Pfa from the sponge *P. ficiformis*. This genome had 6 and 3 copies of the two regulators, respectively, which was in the range of free-living Thaumarchaeota. While *P. ficiformis* is a high microbial abundance sponge (Vacelet and Donadey, 1977; Gloeckner et al., 2014), indicating a dense associated microbial symbiont community, it obtains its symbionts by horizontal transfer (Maldonado and Riesgo, 2009). Therefore, its symbionts likely need to survive outside of the sponge. This could explain why its thaumarchaeal symbiont needs more genes for nitrogen metabolism regulation than the symbionts of other sponge hosts.

Marine planktonic Thaumarchaeota are mixotrophs that can take up organic nitrogen and carbon (Ouverney and Fuhrman, 2000; Herndl et al., 2005; Kirchman et al., 2007). However, recent experimental data suggest that most assimilate only the nitrogen from amino acids rather than assimilating the complete amino acid (Dekas et al., 2019). As expected, we found ABC-type transporters for di- and oligopeptides (based on COG annotations). An amino acid transporter (COG0531) was almost exclusively limited to the free-living Thaumarchaeota. In contrast, an ABC-type branch-chained amino acid transport system was found in ten of the 11 sponge-associated Thaumarchaeota and absent from the genomes analyzed here of free-living Thaumarchaeota. Given that this transporter type can have a wide range of substrates (Hosie et al., 2002), we do not know which amino acids are taken up by the sponge symbionts. Branch-chained amino acid transporters are expressed in the sponge host based on metatranscriptome and -proteome data (Liu et al., 2012; Moitinho-Silva et al., 2017a; Moeller et al., 2019). They can also be found in the genomes of bacterial sponge symbionts (Gauthier et al., 2016). The expression of thaumarchaeal branched-chain amino acid transporters in the sponge host could indicate a mixotrophic lifestyle of its thaumarchaeal symbionts as previously suggested (Moeller et al., 2019), but experiments are necessary to determine if also the carbon is taken up and not only the amino group as shown for free-living Thaumarchaeota (Dekas et al., 2019).

### Vitamin Production

Microbial symbionts of animals are important contributors to their hosts’ metabolism through the production of essential nutrients such as vitamins and cofactors (Moya et al., 2008). Free-living marine Thaumarchaeota are known as vitamin B12 producers (Doxey et al., 2015; Heal et al., 2017). Analysis of the microbial community of the sponge *Ircinia ramosa* identified Thaumarchaeota symbionts as producers of riboflavin (vitamin B2), biotin (vitamin B7), and cobalamin (vitamin B12) (Engelberts et al., 2020). Using the SEED annotations, we found that all 23 Thaumarchaeota analyzed, including the sponge symbionts, have the genetic potential to synthesize pyridoxin (B6) and coenzyme F420. Most sponge-associated Thaumarchaeota could also synthesize the vitamins B1, B2, B7, and B12 (Supplementary Table S12). Two symbionts lacked genes for the synthesis of these vitamins. *Ca*. Cenporiarchaeum stylissum S13 lacked a single gene in the biosynthetic pathways of vitamin B1 and B7, and 5 genes for vitamin B12 synthesis. As it is the least complete genome, we cannot rule out the possibility that it can produce these vitamins. The newly obtained Thaumarchaeon TS lacked two genes for vitamin B2 synthesis, which likewise might be due to incompleteness. Sponge-associated Thaumarchaeota have, therefore, the genetic potential to provide vitamins to their host and its microbial community.

### Functional Differences Between Sponge-Associated and Free-Living Thaumarchaeota

**FIGURE 4:**
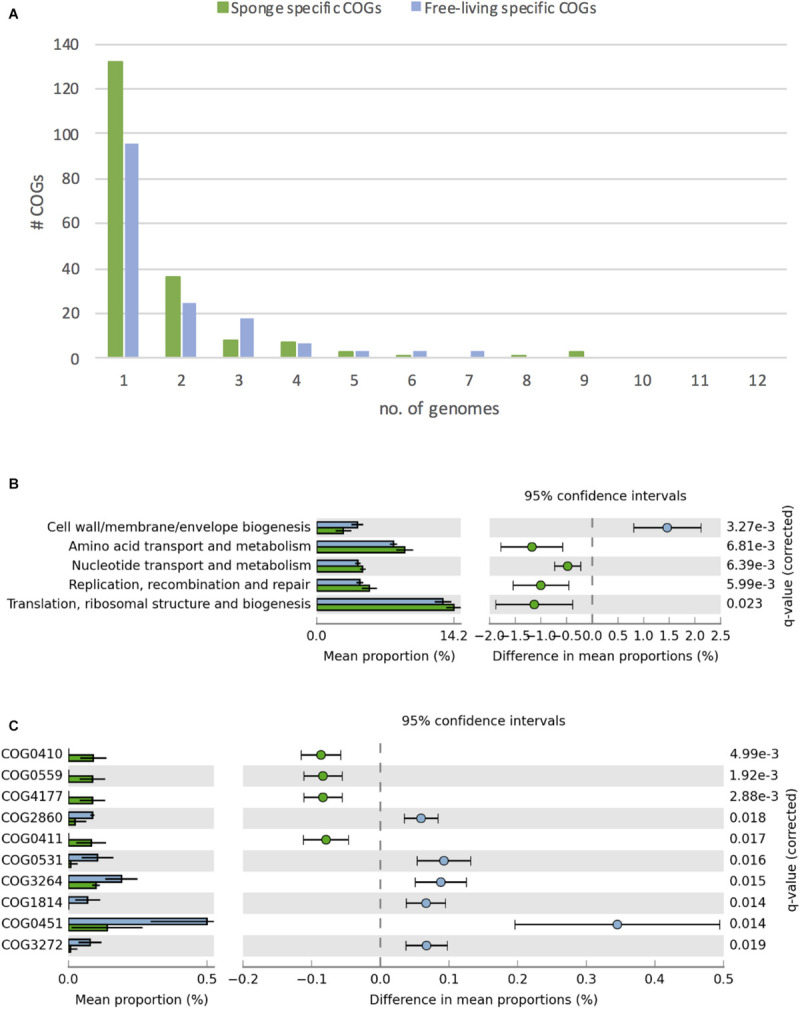
Differences in COGs between free-living (blue) and sponge-associated (green) Thaumarchaeota. **(A)** Distribution of free-living and sponge symbiont specific COGs among genomes. The maximum for free-living Thaumarchaeota is 12 and for sponge-associated ones is 11 genomes. **(B)** COG classes with significant differences in free-living vs. sponge symbiont genomes (*t*-test, FDR Benjamin-Hochberg corrected *p*-value <0.05). **(C)** Distribution of COGs with significant differences (*t*-test, FDR Benjamin-Hochberg corrected *p*-value <0.05). COGs 0410, 0559, 4177, and 0411 are part of the branched chain amino acid transporter. Free-living enriched COGs were related to an amino acid transporter (COG0531), a Fe^2 +^ /Mn^2 +^ transporter (COG1814), a small-conductance mechanosensitive channel (COG2364), a nucleoside-diphosphate-sugar epimerase (COG0451), an uncharacterized membrane protein (COG2860), and a protein of unknown function (COG3272).

Overall, we identified 1394 COGs from 24 classes in the analyzed genomes. 156 COGs were only found in free-living and 191 only in sponge-associated genomes (Supplementary Table S7). Most of the COGs specific to sponge symbionts (88%) were only present in one (132 COGs) or two (36 COGs) genomes. None of these symbiont specific COGs was present in all sponge-associated Thaumarchaeota genomes. A similar trend was found in the free-living Thaumarchaeota with 78% of the free-living specific COGs being present only in one or two genomes and none present in all (Figure 4A).

Sponge-associated genomes were significantly enriched in the COG classes “Replication, recombination & repair,” “Nucleotide transport & metabolism,” “Amino acid transport & metabolism,” “Translation, ribosomal structure & biogenesis,” and significantly depleted in the class “Cell wall/membrane/envelope biogenesis” (t-test, two-tailed, FDR-Benjamin-Hochberg corrected *p*-values <0.05) (Figure 4B). Some of these differences (e.g., the enrichment in “Replication, recombination & repair” and reduction in “Cell wall/membrane/envelope biogenesis”) fit earlier reports from sponge metagenomes (Thomas et al., 2010) and bacterial symbionts (Burgsdorf et al., 2015).

10 COGs were significantly different distributed between sponge symbionts and free-living Thaumarchaeota (*t*-test, FDR corrected *p*-value <0.05) (Figure 4C). The four COGs (COG0410, COG0411, COG0559, COG4177) of the sponge-specific ABC branched-chain amino acid transporter were the only COGs significantly enriched in sponge symbionts. The only sponge symbiont without any part of this transporter was *Ca.* Nitrosopumilus sp. Nsub. The six COGs significantly enriched in free-living Thaumarchaeota included the above-mentioned amino acid transporter (COG0531), which was present in 11 of 12 free-living Thaumarchaeota in 1 to 3 copies and among sponge-associated Thaumarchaeota only in *Ca.* Nitrosopumilus cymbastellus in a single copy. Another transporter limited to most free-living Thaumarchaeota was COG1814, a predicted Fe^2 +^ /Mn^2 +^ transporter of the VIT1/CCC1 family. It was present in 9 of the 12 analyzed free-living Thaumarchaeota genomes (1–2 copies). Other COGs enriched in the free-living Thaumarchaeota included two COGs of class M Cell wall/membrane/envelope biogenesis, COG0451 (a nucleoside-diphosphate-sugar epimerase) and COG3264 (a small-conductance mechanosensitive channel). The former was present in 0–5 copies in sponge symbionts compared to 2–12 copies in free-living ones, whereas the latter had 1 copy in sponge symbionts and 2–3 copies in the free-living ones except for the two pelagic Pacific Ocean Thaumarchaeota, which also had a single copy. Finally, COG2860 and COG3272, for which no function is known (class S), were both enriched in the free-living Thaumarchaeota (Supplementary Table S8).

### Proteins Involved in Host-Symbiont Interactions

Eukaryotic like protein (ELP) domains in the genomes of sponge symbionts have been suggested to be important for the interaction between the symbiont and their hosts (Fan et al., 2012; Hentschel et al., 2012; Reynolds and Thomas, 2016). ELP domains such as ankyrin repeats and tetratricopeptide (TPR) repeats are often enriched in sponge-associated bacteria and sponge metagenomes (e.g., Fan et al., 2012; Burgsdorf et al., 2015). Hence, we investigated if there are ELPs that are enriched in sponge-associated Thaumarchaeota and if there are ELPs that are only present in sponge-associated Thaumarchaeota. We found no common distribution pattern of ELPs in the sponge symbionts. The distribution of several known and some new ELP candidates appeared to be highly specific. ELPs that were exclusively found in sponge-associated Thaumarchaeota, were encoded in up to three sponge symbiont genomes (Supplementary Table S11). Ankyrin repeats, known to be enriched in various sponge-associated bacterial symbionts and sponge metagenomes (Fan et al., 2012; Burgsdorf et al., 2015) and shown to alter phagocytosis of amoeba (Nguyen et al., 2014), were only present in *Ca*. Nitrosopumilus sp. ESC. Other ELP domains previously shown to be enriched in sponge metagenomes (Fan et al., 2012) included leucine-rich repeat DUF285 and PQQ enzyme repeats. These were only present in 2 and 1 thaumarchaeal symbiont genomes, respectively, with the leucine-rich repeat found in two of the novel genomes. We also identified four ELP domains specific to sponge-associated Thaumarchaeota that have not been previously reported from bacterial sponge symbionts. These were the CUB-domain, the EPTP domain, pentatricopeptide repeats (PPR), and annexin. The deep-sea sponge symbionts *Ca*. Nitrosopumilus sp. ESC and LS AOA encoded a protein with a CUB domain. CUB-like domains are widely occurring structural motifs that in eukaryotes are found almost exclusively in extracellular and plasma membrane-associated proteins. They are involved in a wide range of biological functions, including cell signaling, axon guidance and receptor-mediated endocytosis, and are generally involved in the recognition of substrates and binding partners (Blanc et al., 2007). In the *T. swinhoei* symbiont MAG, we identified a genomic sequence that encodes an EPTP domain, which occurs in eukaryotic receptors and secreted proteins (Staub et al., 2002). PPR domains, structurally similar to ELP tetratricopeptide repeats that are well known from bacterial sponge symbionts (e.g., Fan et al., 2012; Burgsdorf et al., 2015), were encoded by *Ca*. Nitrosopumilus detritiferus H13 and *Ca.* Nitrosopumilus cymbastellus. PPRs are common in plant genomes. They play essential roles in posttranscriptional processes in mitochondria and chloroplasts (Lurin et al., 2004). Annexin, which is involved in the endocytosis and exocytosis of vesicles in eukaryotes (Gerke and Moss, 1997), was encoded by *Ca.* Nitrosopumilus cymbastellus.

**FIGURE 5:**
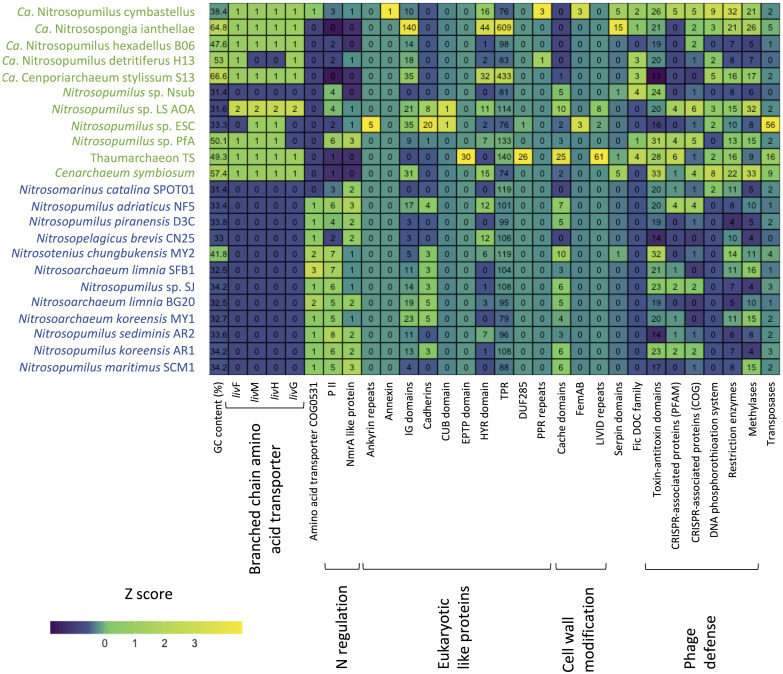
Heatmap summarizing functional differences in the genomic repertoire between sponge-associated (green) and free-living (blue) Thaumarchaeota. Numbers indicate copies per MAG.

Several other ELP domains were found in the genomes of some sponge-associated Thaumarchaeota, but also present in several free-living Thaumarchaeota. These included cadherins, TPRs, NHL repeats, HYR domains, Ig domains, TIR domains and WD40-like repeats (Supplementary Table S11). Fibronectin type III, which was previously found to be enriched in sponge-metagenomes (Fan et al., 2012) and the sponge cyanobacterial symbiont *Ca.* Synechococcus feldmannii (Burgsdorf et al., 2019), and von Willebrand factor type A domains did not show any obvious abundance difference between sponge-associated and free-living Thaumarchaeota. Laminin domains, which are enriched in Poribacteria (Siegl et al., 2011), were present in four sponge-associated Thaumarchaeota but were not enriched compared to free-living Thaumarchaeota. Overall, ELPs are likely to play a role in the recognition of sponge-associated Thaumarchaeota as symbionts by their host sponge. In contrast to their bacterial counterparts, we found high inter-species variability in ELPs, such that each ELP was only shared among a few genomes at most (Figure 5). This suggests Thaumarcheaota employ diverse strategies for interacting with their hosts.

Another proposed way for bacterial sponge symbionts to avoid digestion by their sponge host is the modification of the O-antigen on the cell wall surface. The absence of rhamnose in the sponge-associated cyanobacterium *Ca*. Synechococcus spongiarum has been suggested to aid in the evasion of sponge-phagocytosis and phage predation (Burgsdorf et al., 2015). Thaumarchaeota have surface layer glycoproteins (Rodrigues-Oliveira et al., 2017) and rhamnose can be part of these proteins in archaea (Schäffer and Messner, 2004). Of the analyzed genomes, eight sponge-associated thaumarchaeal genomes missed all four COGs necessary for rhamnose biosynthesis (COG1898 dTDP-4-dehydrorhamnose 3,5-epimerase and related enzymes, COG1091 dTDP-4-dehydrorhamnose reductase, COG1088 dTDP-D-glucose 4,6-dehydratase, COG1209 dTDP-glucose pyrophosphorylase). The other three symbiont genomes (*Ca*. Nitrosopumilus sp. LS AOA, *Ca*. Nitrosopumilus cymbastellus, *Ca*. Nitrosospongia ianthellae) encoded two out of these four COGs. Eleven of the 12 free-living Thaumarchaeota had at least two of these COGs, with only *Ca*. Nitrosomarinus catalina SPOT01 having none (Supplementary Table S8). Hence, the modified surface layer glycoproteins might aid thaumarchaeal symbionts to evade digestion by their host sponges as proposed for *Ca*. Synechococcus spongiarum.

Another six Pfam annotated domains involved in outer cell wall modifications were present only in one to three symbiont genomes and absent from the genomes of free-living Thaumarchaeota. The OmpA domain is known from outer membrane proteins of Gram-negative bacteria. It is necessary for pathogenesis in *E. coli* and can interact with host receptor molecules (Selvaraj et al., 2007). FemAB domains are involved in cell wall synthesis and mutations in them decrease antibiotic resistance in *Streptococcus* strains (Rohrer and Berger-Bächi, 2003). The PEGA domain has been previously found in archaea and is structurally similar to surface-layer proteins (Adindla et al., 2004). Likewise, the LVIVD domain, which was found in three sponge symbionts (Figure 5), especially Thaumarchaeon TS, is a component of bacterial and archaeal surface proteins (Adindla et al., 2004). Two other sponge-specific domain annotations involved in cell wall modifications are related to bacterial O-antigen modifications and might have similar roles in archaeal surface layer proteins. The O-antigen polysaccharide polymerase Wzy links O-units via a glycosidic linage to form a long O-antigen. WbqC-like proteins might be involved in O-antigen production in bacteria. Finally, we found cache domain (3 Pfams, two sponge-specific) to be enriched in some sponge-associated archaea. Cache domains are extracellular domains likely having a role in small-molecule recognition and might be potential chemotaxis receptors (Anantharaman and Aravind, 2000). Given that the six sponge symbiont-specific domains and the enriched cache domains are on the cell surface, they are potential candidates for specific recognition between the sponge-associated Thaumarchaeota and their host sponges and thus might help the symbiont avoid digestion by its host.

Sponge-associated MAGs also encoded proteins that can potentially modulate the host metabolism and the immune system. Seven of the 11 sponge symbionts and none of the free-living Thaumarchaeota had proteins with a FIC domain (PF02661) (Figure 5). This domain family is widespread in bacteria and has multiple functions including the toxin component of toxin-antitoxin systems. In pathogenic bacteria, the FIC domain bearing toxins can divert host cell processes (Veyron et al., 2018). The FIC domain was overrepresented in sponge metagenomes compared to seawater metagenomes (Fan et al., 2012). Previous work identified 15 genes in *Ca*. Nitrosospongia ianthellae encoding serpin domains (Moeller et al., 2019). Serpins belong to a class of irreversible substrate inhibitors of proteases, often of the serine class. Here, we show that the domain was also present in the genomes of the sponge symbionts *Cenarchaeum symbiosum* (5 copies), *Ca*. Nitrosopumilus cymbastellus (5 copies), Thaumarchaeon TS (1 copy), *Ca*. Nitrosopumilus sp. Nsub (1 copy), while among free-living Thaumarchaeota, only the soil-inhabiting *Nitrosotenuis chungkubensis* MY2 had a single copy (Figure 5). Serine proteases are found as part of the innate immune system in some sponges (Riesgo et al., 2014) and are known activators of the lectin complement pathway, a highly sophisticated host defense system that detects, contains and kills pathogens (Iwaki et al., 2011). In gut bacteria, serpins have been suggested to act as protection against exogenous proteolysis (Ivanov et al., 2006). Therefore, symbiotic Thaumarchaeota might use serpins as protection against digestion by sponge amoebocytes, the “feeding cells” of sponges, with which they must coexist within the sponge mesohyl matrix.

### Defense Against Phages

Due to the high filtration activity of their host, sponge symbionts are likely exposed to a vast number of phages (Hadas et al., 2006; Jahn et al., 2019). Sponge metagenomes and genomes of bacterial symbionts are often enriched in genes involved in the defense against phages such as CRISPR-Cas, restriction-modification systems, and toxin-antitoxin modules (Fan et al., 2012; Horn et al., 2016; Podell et al., 2019). This does not seem to be the case in sponge-associated Thaumarchaeota. COG class V “Defense mechanisms” did not differ significantly between the free-living and sponge-associated Thaumarchaeota analyzed. While we found components of several toxin-antitoxin modules, sponge-associated Thaumarchaeota genomes were overall not enriched in toxin-antitoxin modules, when compared to free-living Thaumarchaeota ones (irrespective of whether COG or Pfam annotations were used, both two-tailed *t*-test, *p* > 0.05) (Supplementary Tables S8, S11, respectively). Some modules were present only in sponge-associated Thaumarchaeota genomes. This included a putative abortive infection system (AbiE family), which has only recently been reported in archaea (Li et al., 2018) (Supplementary Table S11). However, another domain, an ATPase domain associated with the cellular activity (AAA) (PF13304), which in some cases has been associated with the Abi toxin, was found in all genomes in varying numbers (Supplementary Table S11).

While bacterial sponge symbionts are often enriched in CRISPR-associated genes compared to closely related free-living bacteria (Fan et al., 2012; Horn et al., 2016), this is not clear in sponge-associated Thaumarchaeota. Sponge-associated Thaumarchaeota did not differ significantly from free-living Thaumarchaeota in cas domains when using Pfam annotations (two-tailed *t*-test, *p* = 0.31), but were enriched in CRISPR-associated genes when using COG annotations (two-tailed *t*-test, *p* = 0.047). Irrespective of COG or Pfam annotation, all free-living Thaumarchaeota had between 0 and 2 CRISPR-associated genes except for *Nitrosopumilus adriaticus* NF5, which had 4. The sponge-associated Thaumarchaeota *Ca*. Nitrosopumilus sp. PfA, *Ca*. Nitrosopumilus sp. LS AOA, *Ca*. Nitrosopumilus cymbastellus, and, depending if COG or Pfam annotations were used either *Cenarchaeum symbiosum* or Thaumarchaeon TS had 4–6 CRISPR-associated genes surpassing most free-living Thaumarchaeota (Figure 5 and Supplementary Tables S8, S11, respectively). Hence enrichment in CRISPR-associated genes is present in some Thaumarchaeota, but not as common as it appears to be for bacterial sponge symbionts [but see recent work on Verrucomicrobia for an exception (Sizikov et al., 2020)].

The DNA phosphorothioation system, which in archaea likely works as a defense system against phage (Xiong et al., 2019), appeared to be more prevalent in sponge-associated than free-living Thaumarchaeota. In addition to the previously reported presence in *Ca*. Nitrosospongia ianthellae and *Ca*. Nitrosopumilus cymbastellus (Moeller et al., 2019), we found at least one of *dndB* and *dndE* in the Thaumarchaeon TS and in *Ca*. Cenporiarchaeum stylissum S13 MAGs. Only one free-living Thaumarchaeota species, *Ca*. Nitrosomarinus catalina SPOT01, encoded this system (Ahlgren et al., 2017). When including the protein domains DUF262 (PF03235) and DUF1524 (PF07510), which have been previously found in *dndB* genes and identified as components of restriction-modification systems (Makarova et al., 2011; Moeller et al., 2019), four more sponge symbionts were found to have at least part of the system: *Cenarchaeum symbiosum*, *Ca*. Nitrosopumilus sp. ESC, *Ca*. Nitrosopumilus sp. LS AOA and *Ca*. Nitrosopumilus detritiferus H13. Metagenome analyses showed that the DUF262 and DUF1524 domains are enriched in sponge metagenomes (Fan et al., 2012; Horn et al., 2016).

The sponge-associated Thaumarchaeota were also enriched in restriction enzymes (two-tailed *t*-test, *p* = 0.006). Nine of the 19 different Pfam domains related to restriction enzymes were found only in the sponge-associated Thaumarchaeota, whereas two domains were specific to the free-living Thaumarchaeota. The same significant difference was found when analyzing domains of methylases and methyltransferase known to be involved in restriction modifications systems (two-tailed *t*-test, *p* = 0.037). Here three of nine domains were specific to sponge symbionts. The number of domains of both elements of the restriction-modification system was significantly correlated (Pearson’s *r* 0.7007, *p* < 0.001). The enrichment of restriction-modification systems in some sponge-associated Thaumarchaeota was noted previously (Moeller et al., 2019; Zhang et al., 2019). This enrichment is also seen in bacterial members of sponge microbial communities relative to free-living bacteria (Horn et al., 2016; Slaby et al., 2017; Burgsdorf et al., 2019), which points to the importance of phage defense mechanisms within the sponge host for both these microbial domains.

### Potential for Horizontal Gene Transfer

Many bacterial sponge symbionts are enriched in transposases (Fan et al., 2012), which suggests enhanced horizontal gene transfer between members of the sponge microbiome (Pita et al., 2018). This does not seem to be the case for the majority of sponge-associated Thaumarchaeota, as the mobilome COG class X did not differ significantly between sponge-associated and free-living Thaumarchaeota. However, 11 of the 14 transposases (or inactive derivatives) annotated by COG and 13 out of 16 domains annotated by Pfam were restricted to the sponge symbiont genomes. Sponge-associated Thaumarchaeota had a larger variability in transposases ranging from 0 to 21 COGs and 0 to 56 Pfam domains per genome, compared to only 0–3 in genomes of free-living ones (with the exception of the soil-inhabiting *Nitrosotenius chungbukensis* MY2) (Figure 5 and Supplementary Tables S8, S11). Based on COG annotations, the three newly analyzed genomes and *Ca*. Nitrosopumilus cymbastellus had more transposases than their marine free-living counterparts ranging from 5 COG annotated transposases in *Ca*. Nitrosopumilus cymbastellus to 21 in Thaumarchaeon TS (Supplementary Table S8). Pfam annotations but not COG annotations confirmed the previously reported enrichment of transposases in *Ca*. Nitrosopumilus ianthallae and *C. symbiosum* (Moeller et al., 2019) (Supplementary Tables S8, S11). Together this indicates a greater potential for gene transfer in some thaumarchaeal sponge symbionts, though it remains unclear why the high number of transposases is restricted to these few sponge-associated thaumarchaeal genomes.

### Metabolic Functions Specific to the Novel Sponge Archaea

The two novel sponge-associated Thaumarchaeota genomes *Ca*. Nitrosopumilus sp. ESC and Thaumarchaeon TS harbored the glycine cleavage system, which was absent from all other genomes. It consisted of the genes *gcvHPT* [COG0509, COG0403/COG1003, COG0404 and COG1249, the dihydrolipoamide dehydrogenase (E3)] together with the genes necessary for lipoate production (COG0095, COG0320), which binds to GcvH. The system enables the reversible reaction glycine + tetrahydrofolate (THF) + NAD^+^ ↔ 5,10-methylene-THF + CO_2_ + NH_3_ + NADH + H^+^. It has been recently found in the genomes of free-living deep-sea Thaumarchaeota (León-Zayas et al., 2015; Wang et al., 2019; Zhong et al., 2020). The methylene-THF can be used in the biosynthesis of purine and methionine (León-Zayas et al., 2015). When working in the direction of glycine cleavage, the reaction provides energy. Moreover, via the downstream oxidation of ammonia and regeneration of THF, it would enable an additional way to generate energy for the sponge symbionts (Figure 3).

Out of the 23 analyzed genomes, the Thaumarchaeon TS from the Red Sea sponge *T. swinhoei* and *Ca*. Nitrosopumilus sp. PfA from *P. ficiformis* collected from the Southeastern Mediterranean Sea were the only ones that encoded exopolyphosphatases (COG0248). This in addition to a high-affinity phosphate transporter consisting of PhoU, PstSABC, which was also present in six other sponge symbionts and six free-living Thaumarchaeota [based on SEED annotations (Supplementary Table S13)]. Phosphorus sequestration in the form of polyphosphate by microbial symbionts has been shown in Caribbean sponges (Zhang et al., 2015). In the case of the symbiont of *P. ficiformis*, the sponge was collected in the Southeastern Mediterranean Sea, which is known for phosphorus limitation (Krom et al., 1991). It is possible that *Ca*. Nitrosopumilus sp. PfA has a free-living phase (see above), during which it experiences phosphorus stress.

## Conclusion

Free-living marine Thaumarchaeota are known as ammonium oxidizers and are key suppliers of vitamins in microbial communities. Our comparative genome analysis supports the previous notion that these traits are conserved in sponge-associated Thaumarchaeota. Some genomic features known to be enriched in bacterial sponge symbionts were also enriched in sponge-associated archaea (e.g., restriction-modification systems), suggesting that the sponge environment selects for these features. However, adaptations to life inside the sponge host appear to diverge in Thaumarchaeota symbionts, as many of these traits were specific only to a few symbiont genotypes. For example, we found a glycine cleavage system and polyphosphatases only in the three novel symbiont genomes. Thus, the analysis of additional novel Thaumarchaeota symbionts is likely to reveal more unique adaptations to the symbiosis with sponges.

## Data Availability Statement

The datasets presented in this study can be found in online repositories. The names of the repository/repositories and accession number(s) can be found in the article/Supplementary Material.

## Author Contributions

MH, IB, and LS conceived the study. IB and MR-B collected samples and isolated DNA for metagenome sequencing. MH, IB, KH, and MR-B did bioinformatics analyses. MH and LS wrote the manuscript with the contribution of all the authors. All authors contributed to the article and approved the submitted version.

## Conflict of Interest

The authors declare that the research was conducted in the absence of any commercial or financial relationships that could be construed as a potential conflict of interest.

## Footnotes

1 http://hannonlab.cshl.edu/fastx_toolkit/

2 http://sourceforge.net/projects/bbmap/

3 www.earthmicrobiome.org

4 https://jgi.doe.gov/data-and-tools/bbtools/

5 https://img.jgi.doe.gov/cgi-bin/mer/main.cgi
