# Supplementary material for: Genomic Insights Into the Lifestyles of Thaumarchaeota Inside Sponges
Source: Front Microbiol. 2021 Jan 11;11:622824. doi: 10.3389/fmicb.2020.622824 (PMC7848895; doi:10.3389/fmicb.2020.622824)
Supplement: Supplementary file 1 [file Data_Sheet_1.PDF]

Supplementary Information

Archaeal 16S rRNA recovery from the sponge Petrosia ficiformis

We generated a targeted small subunit rRNA gene assembly from the *P. ficiformis* metagenome using EMIRGE (Miller et al., 2011). EMIRGE was run with 100 iterations, followed by read mapping to a de-replicated version of the SILVA 108 SSU rRNA database. During each iteration, sequences were merged into OTUs of ≥97% similarity. The reconstructed 16S rRNA gene sequences were then classified with the classification tool of the SINA aligner v.1.2.11 (Pruesse et al., 2012). Two thaumarchaeal sequences were recovered. One was a near full-length sequence (1249 bp) with 61 bp gap in the middle, and the other was a shorter one (441 bp). Based on the coverage of the genomes and the relative abundance inferred by EMIRGE, we assigned the longer one to bin A.

As the EMIRGE sequence did not cover the part used in the Sponge Microbiome Project, we created a 16S rRNA clone library from the same *P. ficiformis* DNA as used for metagenomic sequencing. The library was created with archaea specific primers modified for enhanced amplification of marine group I.1a Thaumarchaeota. Modifications in the primer sequence are indicated in bold. The PCR mix consisted of 1 µl 10 µM of each modified version of Arch21F (DeLong, 1992) (5'-**A**TCCGGTTGATCC**T**GCCGGA) and Arch1492R (Teske et al., 2002) (5'-GG**TC**ACCTTGTTACGACTT), 25 µl 2x RedTaq Readymix, 21 µl molecular grade water and 1 µl DNA template. Amplification was performed with the following conditions: an initial denaturation for 5min at 94°C; 35 cycles of 40s at 94°C, 30s at 56°C, 40s at 72°C; a final extension of 10min at 72°C. The PCR product was extracted from a 1% agarose gel in TAE buffer using the QIAquick gel extraction kit following the manufacturer's instructions. For ligation, 3 µl of the purified PCR product were mixed on ice with 5 µl 2 x Rapid ligation buffer, 1 µl pGEM-T easy plasmid (50ng), 1 µl T4 ligase (3U/µl) and incubated at 25°C for 90 min. Heat-shock competent *Escherichia coli* JM109 cells were transformed with the plasmid. The plasmids of 10 white colonies with inserts of the right size were purified and sequenced at Macrogen South Korea using both primers (SP6 and T7). For each colony, a single, combined sequence was obtained after removing the plasmid and 16S rRNA primer parts and the ten resulting sequences were used in the 16S rRNA gene phylogenetic analysis in addition to the EMIRGE sequence.

Supplementary figure

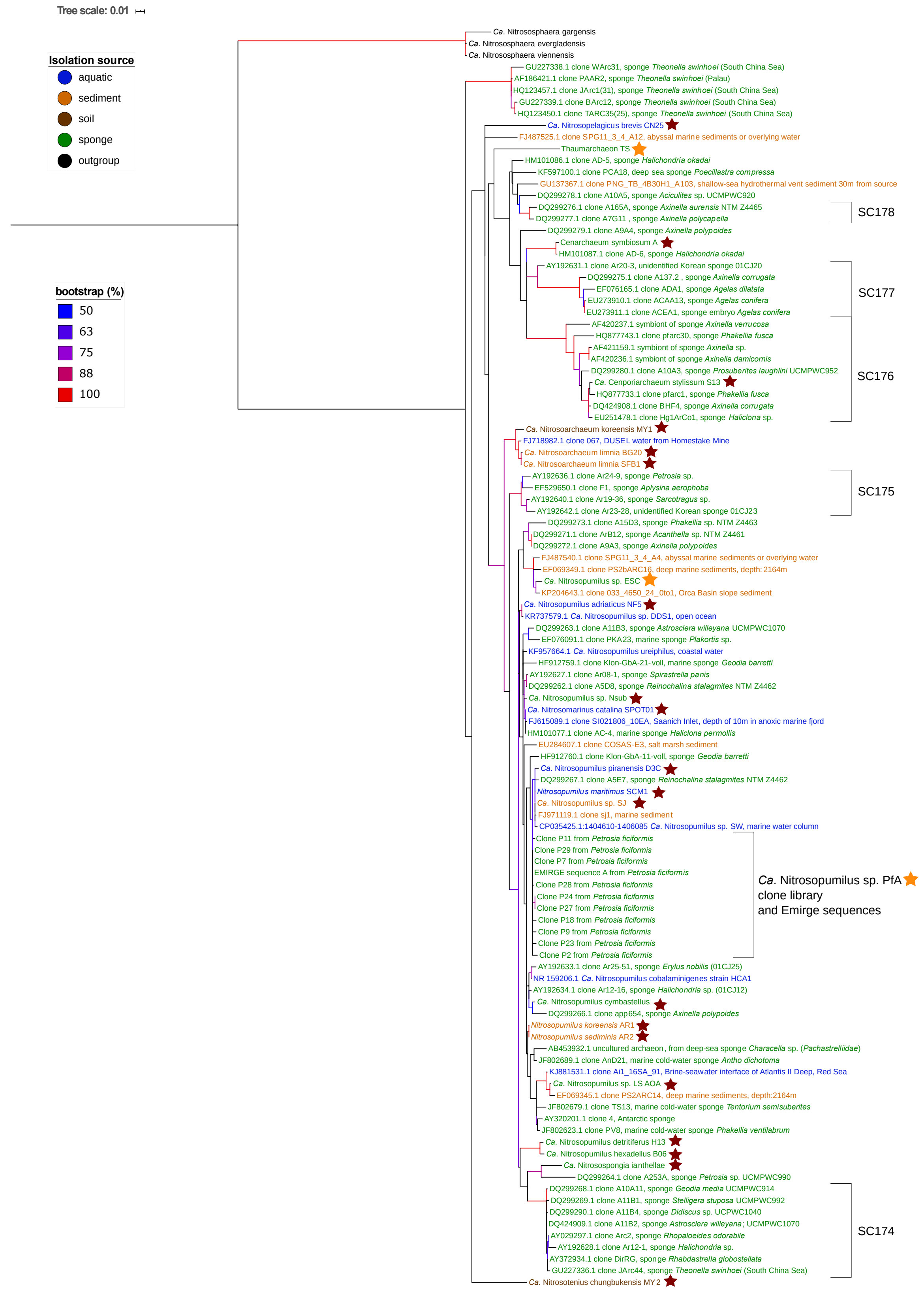

Supplementary figure S1: 16S rRNA gene tree of Thaumarchaeota from sponges (green), marine sediment (orange), seawater (blue) and soil (brown). Sequences from analyzed genomes are indicated by a star. Orange filled stars indicate novel genomes obtained in this study. Branch color indicates bootstrap support. Sponge-specific cluster (SC) from Simister et al. 2012 are indicated. All obtained sequences from *Petrosia ficiformis* from this study group together. The tree was calculated using maximum likelihood with the Kimura-2-parameter substitution model with a portion of invariant sites and a gamma-shaped distribution of mutation rates. Sites with >5% ambiguous or missing data were omitted. Details of the analyses are given in the method section of the article. Different branch colors indicate differences in bootstrap support based on 500 replicates.

References in Supplementary material

DeLong, E. F. (1992). Archaea in coastal marine environments. *PNAS* 89, 5685–5689. doi:10.1073/pnas.89.12.5685.

Miller, C. S., Baker, B. J., Thomas, B. C., Singer, S. W., and Banfield, J. F. (2011). EMIRGE: reconstruction of full-length ribosomal genes from microbial community short read sequencing data. *Genome Biology* 12, R44. doi:10.1186/gb-2011-12-5-r44.

Pruesse, E., Peplies, J., and Glöckner, F. O. (2012). SINA: Accurate high-throughput multiple sequence alignment of ribosomal RNA genes. *Bioinformatics* 28, 1823–1829. doi:10.1093/bioinformatics/bts252.

Simister, R. L., Deines, P., Botté, E. S., Webster, N. S., and Taylor, M. W. (2012). Sponge-specific clusters revisited: a comprehensive phylogeny of sponge-associated microorganisms. *Environmental Microbiology* 14, 517–524. doi:10.1111/j.1462-2920.2011.02664.x.

Teske, A., Hinrichs, K.-U., Edgcomb, V., Gomez, A. de V., Kysela, D., Sylva, S. P., et al. (2002). Microbial Diversity of Hydrothermal Sediments in the Guaymas Basin: Evidence for Anaerobic Methanotrophic Communities. *Appl. Environ. Microbiol.* 68, 1994–2007. doi:10.1128/AEM.68.4.1994-2007.2002.
